# European Stroke Organisation survey on reperfusion therapy practices

**DOI:** 10.1093/esj/aakag113

**Published:** 2026-09-18

**Authors:** Michele Marvardi, Ronen Leker, Sumanjit Gill, Volker Pütz, Theresa Ullberg, Pierre Seners, Maria Hernández-Pérez, Jonathan M Coutinho, Kristian Karlović, Klaudia Soltesova, Julian Bösel, Valeria Caso

**Affiliations:** Department of Emergency and Emergency Medicine, Gubbio-Gualdo Tadino Hospital, USL Umbria 1, Italy; Department of Neurology, Hadassah-Hebrew University Medical Center, Ein Kerem Campus, Jerusalem, Israel; University College London (UCL) Queen Square Institute of Neurology, London, United Kingdom; Department of Neurology, Faculty of Medicine and University Hospital Carl Gustav Carus, Technische Universität Dresden, Dresden, Germany; Dresden Neurovascular Center, Faculty of Medicine and University Hospital Carl Gustav Carus, Technische Universität Dresden, Dresden, Germany; Department of Neurology and Interdisciplinary Rehabilitation, Klinik Bavaria, Kreischa, Germany; Department of Neurology, Skane University Hospital, Lund University, Lund, Sweden; Neurology Department, Rothschild Foundation Hospital, Paris, France; Institute of Psychiatry and Neuroscience of Paris, INSERM U1266, Université Paris Cité, Paris, France; Stroke Unit, Neurosciences Department, Germans Trias i Pujol University Hospital, Universitat Autònoma de Barcelona, Badalona, Spain; Department of Emergency and Emergency Medicine, Amsterdam University Medical Center, Amsterdam, The Netherlands; Radiology Department, University Clinical Hospital of Mostar, and School of Medicine, University of Mostar, Mostar, Bosnia and Herzegovina; Department of Neurology, Louis Pasteur University Hospital Košice, Mostar, Slovakia; Department of Neurology, University Hospital Heidelberg, Heidelberg, Germany; Neurology Faculty, Johns Hopkins University Hospital, Baltimore, MD, United States; Department of Neurology, Friedrich-Ebert-Krankenhaus Neumünster, Neumünster, Germany; Neurology–Stroke Unit, Saronno Hospital, ASST Valle Olona, Saronno, Italy

**Keywords:** acute stroke, prehospital pathway, thrombectomy, thrombolysis

## Abstract

**Introduction:**

Rapid reperfusion remains critical in acute ischaemic stroke, yet substantial prehospital delays persist. Clinicians across Europe widely use stroke code protocols and reperfusion strategies. However, clinicians lack routine data in clinical practice on prehospital triage, workflow organisation and performance monitoring.

**Patients and methods:**

The European Stroke Organisation (ESO) Endovascular Task Force conducted a multinational, cross-sectional, scenario-based e-survey. The survey targeted emergency medical services (EMS), primary stroke centres (PSCs) and comprehensive stroke centres (CSCs). It evaluated how teams assess patients in the prehospital setting, select transport methods, organise workflows, monitor time intervals and audit practices in European stroke networks.

**Results:**

A total of 296 responses from 43 European countries were analysed. Most originated from urban settings and CSCs. Stroke code activation and hospital pre-notification occurred in over 90% of EMS scenarios. Among EMS respondents, 80.4% transported patients with mild-to-moderate symptoms to the nearest hospital capable of intravenous thrombolysis. However, in cases with suspected LVO, transport destinations varied: 49.0% were transported to PSCs, 31.4% were transported to CSCs and 19.6% were transported to the nearest hospital regardless of reperfusion capability. Monitoring of key time intervals was inconsistent. Emergency medical services did not monitor call-to-hospital arrival times in 25.5% of services. Only 46.7% of PSCs and 53.5% of CSCs routinely monitored EMS arrival times. At the PSC level, 25.0% did not monitor door-in–door-out time, and 30.0% reported dedicated stroke teams were unavailable during nights and weekends. At the CSC level, admission-to-reperfusion time was monitored in 90.8% of centres, but only 28.6% audited delays when they occurred. Additionally, 42.2% of CSCs reported no on-site neuro-interventional team availability during nights or weekends. Fewer than two-thirds of EMS and PSC services, and about one-third of CSCs, reported regular audit activity.

**Conclusions:**

Although stroke code activation and pre-notification are widely implemented across Europe, this survey identifies important gaps in systematic time monitoring and audit practices, alongside a lack of continuous (24/7) specialist coverage across the stroke care pathway. Key performance metrics are frequently not tracked, and audit-driven quality improvement remains limited.

## Graphical Abstract

**Graphical Abstract:**
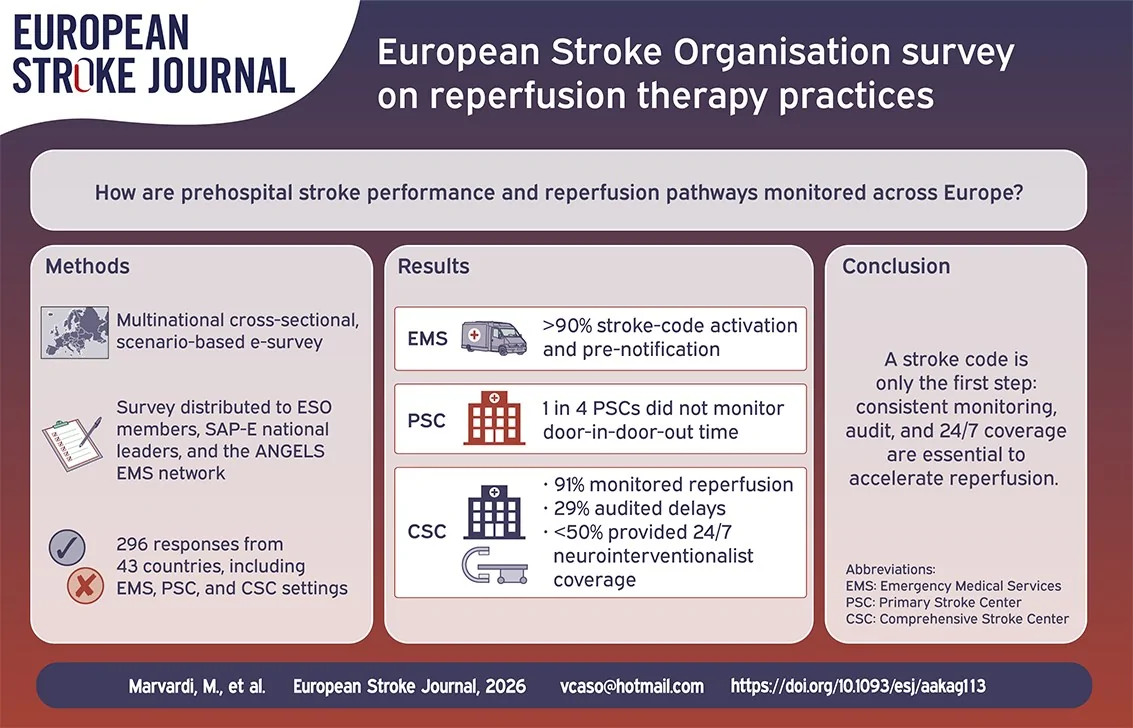

## Introduction

Treatment of stroke is time-dependent, where every minute counts.^1^ In all suspected cases of acute stroke, the primary objectives are to confirm the diagnosis as quickly as possible and initiate reperfusion treatment before irreversible brain injury occurs. This strategy has proven to be the most cost-effective approach to reduce the burden of stroke disease, significantly increasing quality-adjusted life years.^2^ Timely access to high-quality care is crucial: patients who are rapidly admitted to organised stroke centres have a significantly lower 30-day mortality.^3^ In recent years, door-to-needle time (DNT) for thrombolysis and door-to-puncture time for thrombectomy have been significantly reduced.^4^ Numerous studies have in fact demonstrated that shorter DNT is associated with better outcomes at discharge, lower 1-year mortality and readmission rates and better long-term functional outcomes among patients also undergoing EVT.^5^ Despite the progress in in-hospital transport processes, there are often still substantial delays in the pre-hospital phase. Fewer than 10% of patients with acute ischaemic stroke arrive at the hospital within 60 min of symptom onset. Data from the Swedish Stroke Registry show that arrival within 4.5 h occurs in 35%, with no improvement over the last decade.^6^ Less than 20% meet the criteria to receive intravenous thrombolysis (IVT), and only 7.3% and 1.9% receive IVT and EVT, respectively. Among patients receiving reperfusion therapy, the median onset-to-treatment time is approximately 140 min in Western Europe and 150 min in Eastern Europe, exceeding the targets set by the ESO Action Plan 2018-2030, which aim to reduce median onset-to-needle time to < 120 min for IVT and onset-to-reperfusion time to < 200 min for EVT.^7^

The main reasons that potentially eligible patients are refrained from IVT treatment include delayed hospital arrival, absence of stroke-trained personnel on-site and lack of immediate access to brain imaging and laboratory facilities. Endovascular treatment availability is further limited by shortages in trained personnel, infrastructure and funding,^6^ all of which contribute to increased disability and mortality rates.^8^

Two principal pre-hospital stroke triage models are currently used, each with inherent logistical challenges: the “Mothership” (MS) and the “Drip-and-Ship” (DS) models. In the MS model, patients with suspected eligibility for reperfusion therapy are transported directly to a CSC, where both IVT and EVT can be delivered. In contrast, the DS model involves initial transport to the nearest primary stroke centre (PSC) for IVT, followed by secondary transfer to a CSC for EVT when indicated.

However, implementing these models across Europe is limited by access to EVT-capable centres. Only about one-third of countries have enough CSC capacity to meet EVT demands, and in two-thirds of countries, the density of EVT-capable CSCs is below one per million inhabitants, creating significant challenges for timely endovascular care.^9^^,^^10^

Within this framework, the ESO Endovascular Task Force aims to evaluate EMS call-to-arrival intervals in stroke cases and assess how endovascular reperfusion is organised, implemented and monitored in routine clinical practice across Europe. The ultimate goal is to identify, analyse and address the main systemic barriers to improving stroke care delivery and outcomes.

## Patients and methods

After a structured and methodical discussion within the ESO Endovascular Committee—who represent our community of practice in stroke care—the decision was made to design a comprehensive survey. The e-survey was created using Google Forms and was coordinated by the Endovascular Task Force. The survey design process involved reflective discussions and collaborative reviews. Initial drafts were refined based on feedback from committee members and external collaborators. After reaching a consensus on the final structure and content, the survey received formal approval from the ESO Executive Committee.

The survey was distributed via email by the ESO office to national coordinators of the Stroke Action Plan for Europe (SAPE), with additional support from the Angels group to EMS physicians, part of the Angel EMS group and through committee members’ professional networks in their home countries which comprised ESO members (approximately 4000), including SAPE National Stroke Leaders (approximately 120), and to approximately 400 EMS physicians from the ANGELS network.^11^

For the purposes of this survey, the term “European” was used to refer to countries participating in the SAPE and related ESO implementation networks, rather than being restricted to European Union member states or to a narrow geographic definition of Europe.^7^^,^^12^

The complete survey—including all questions and response options—is available in the supplementary file (Figure S1). All data were stored in an online database.

The survey was divided into 2 parts. The first part collected general information about participants’ work activities, including the country of practice, professional role (eg, paramedic/ambulance nurse, stroke nurse, neurologist, neuro-interventionalist, emergency room physician, stroke physician), type of setting (eg, emergency medical services [EMS], PSC, CSC) and environment (urban, suburban, rural).

The second part comprised scenario-based questions directed at staff from EMS, PSCs and CSCs. For the survey, PSCs were defined as dedicated, clearly defined hospital areas or wards where stroke patients receive care from a multi-professional team—including medical, nursing and therapy staff—trained in stroke care and cerebral function, with clearly defined roles, regular interdisciplinary interaction and stroke leadership.^7^ Later in the text, IVT-capable hospital and PSC are synonymous.

Comprehensive stroke centres were defined as hospitals that offer the full continuum of stroke care, including pre-hospital services, acute management, rehabilitation, secondary prevention and access to neurosurgical and endovascular interventions.^7^

## Results

A total of 296 survey responses were analysed, with the largest contributions from Spain (18.9%), Italy (12.8%), Portugal (6.1%) and the Netherlands (5.4%) (Table 1). Respondents were predominantly neurologists (59.1%), followed by stroke physicians (9.1%) and emergency physicians (6.1%). Most responses originated from urban settings (86.1%), with CSCs representing the most common institutional setting (62.5%).

**Table 1:** List of participating nationalities.

European country	*n* (296)	% (100)
**Spain**	56	18.9
**Italy**	38	12.8
**Portugal**	18	6.1
**Netherlands**	16	5.4
**UK and Northern Ireland**	16	5.4
**France**	13	4.4
**Ukraine**	12	4.0
**Sweden**	11	3.7
**Greece**	10	3.4
**Ireland**	10	3.4
**Denmark**	7	2.4
**Israel**	7	2.4
**Romania**	6	2.0
**Czech Republic**	5	1.7
**Serbia**	5	1.7
**Azerbaijan**	4	1.4
**Belgium**	4	1.4
**Bosnia and Herzegovina**	4	1.4
**Croatia**	4	1.4
**Georgia**	4	1.4
**North Macedonia**	4	1.4
**Poland**	4	1.4
**Bulgaria**	3	1.0
**Montenegro**	3	1.0
**Slovakia**	3	1.0
**Türkiye**	3	1.0
**Armenia**	2	0.7
**Belarus**	2	0.7
**Estonia**	2	0.7
**Finland**	2	0.7
**Germany**	2	0.7
**Hungary**	2	0.7
**Iceland**	2	0.7
**Kazakhstan**	2	0.7
**Malta**	2	0.7
**Austria**	1	0.3
**Republic of Cyprus**	1	0.3
**Latvia**	1	0.3
**Lithuania**	1	0.3
**Luxembourg**	1	0.3
**Moldova**	1	0.3
**Russian Federation**	1	0.3
**Uzbekistan**	1	0.3

### Emergency medical services (EMS)

**Figure 1:**
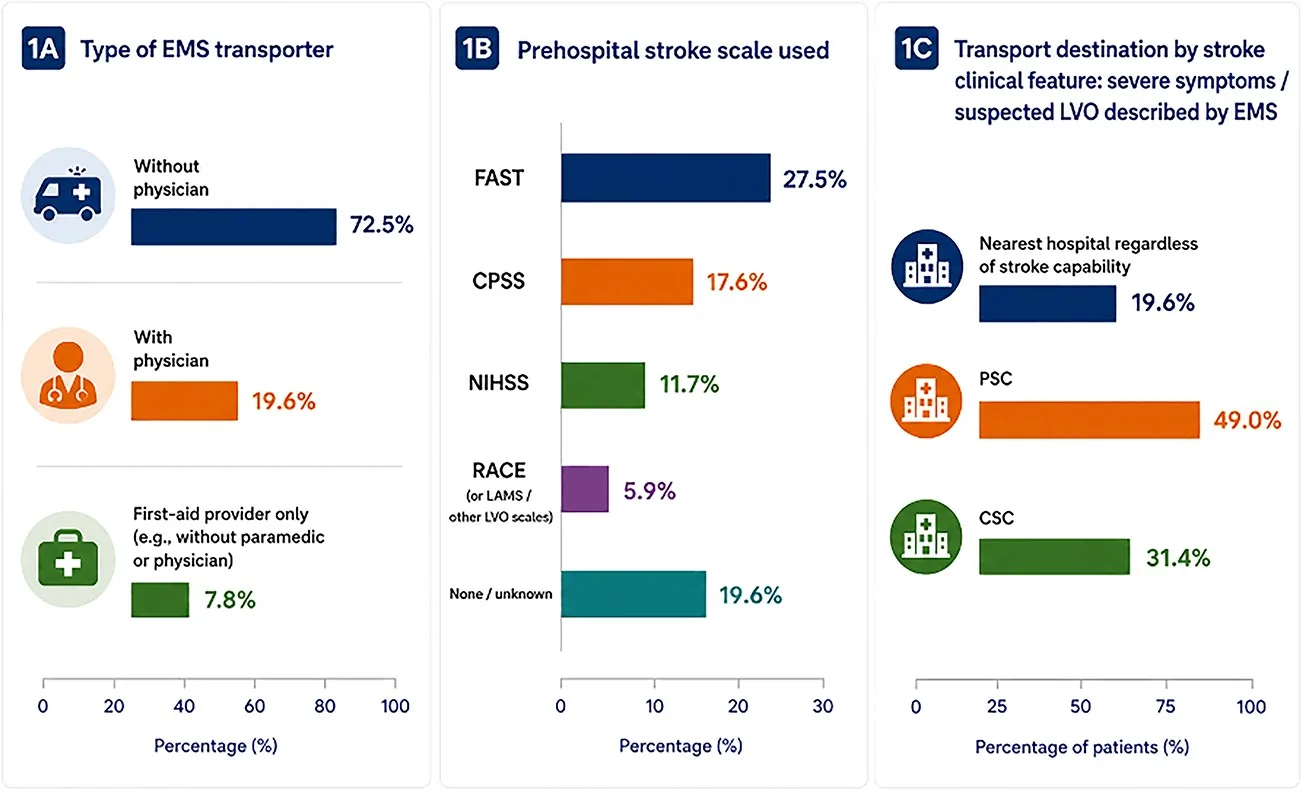
Prehospital stroke care and EMS transport pathways. Panel A shows the type of EMS transporter; panel B reports the prehospital stroke scales used and panel C shows transport destination for patients with severe symptoms or suspected LVO. Abbreviations: CPSS = Cincinnati Prehospital Stroke Scale; CSC = comprehensive stroke centre; EMS = emergency medical services; FAST = face arm speech time; PSC = primary stroke centre; RACE = Rapid Arterial oCclusion Evaluation.

Fifty-one responses (17.2%) were obtained from EMS personnel. In only 19.6% of cases a physician was present during transport of a suspected stroke patient; in 72.5%, transport was handled solely by a paramedic or ambulance nurse. In our study group, Mobile Stroke Units were universally unavailable (Figure 1A).

Pre-hospital stroke assessment varied: 27.5% reported using the FAST scale and 17.6% the Cincinnati Prehospital Stroke Scale (CPSS), while nearly 20% reported not using any prehospital stroke scale (Figure 1B).

Upon EMS arrival and stroke suspicion, over 90% activated a stroke code protocol, and 94% notified the target hospital in advance.

Transport destinations varied by symptom severity. For patients with mild to moderate symptoms, 80.4% were transported to the nearest IVT-capable hospital. For severe symptoms suggestive of LVO, patients were transported to a PSC in 49.0%, a CSC in 31.4% and the nearest hospital irrespective of reperfusion capability in 19.6%. Transport was reported to be slower when the patient was deemed ineligible for reperfusion (Figure 1C).

Patients were most frequently dropped off in the emergency room, 58.8% for mild to moderate symptoms, 52.9% for severe. Direct access to the imaging unit was reported in 29.4% and 33.3% respectively, and only 2% of patients with severe symptoms were taken directly to the angio-suite. For patients not eligible for reperfusion, over 80% were discharged directly to the emergency room.

First-call-to-hospital-arrival time was not monitored in 25.5%, and only 64.7% reported conducting performance audits.

### Primary stroke centres (PSCs)

Sixty responses (20.3%) came from PSC personnel. Only 46.7% systematically monitored the EMS call-to-hospital arrival time, while in 23.3% of cases, no stroke code was activated despite pre-notification by EMS.

**Figure 2:**
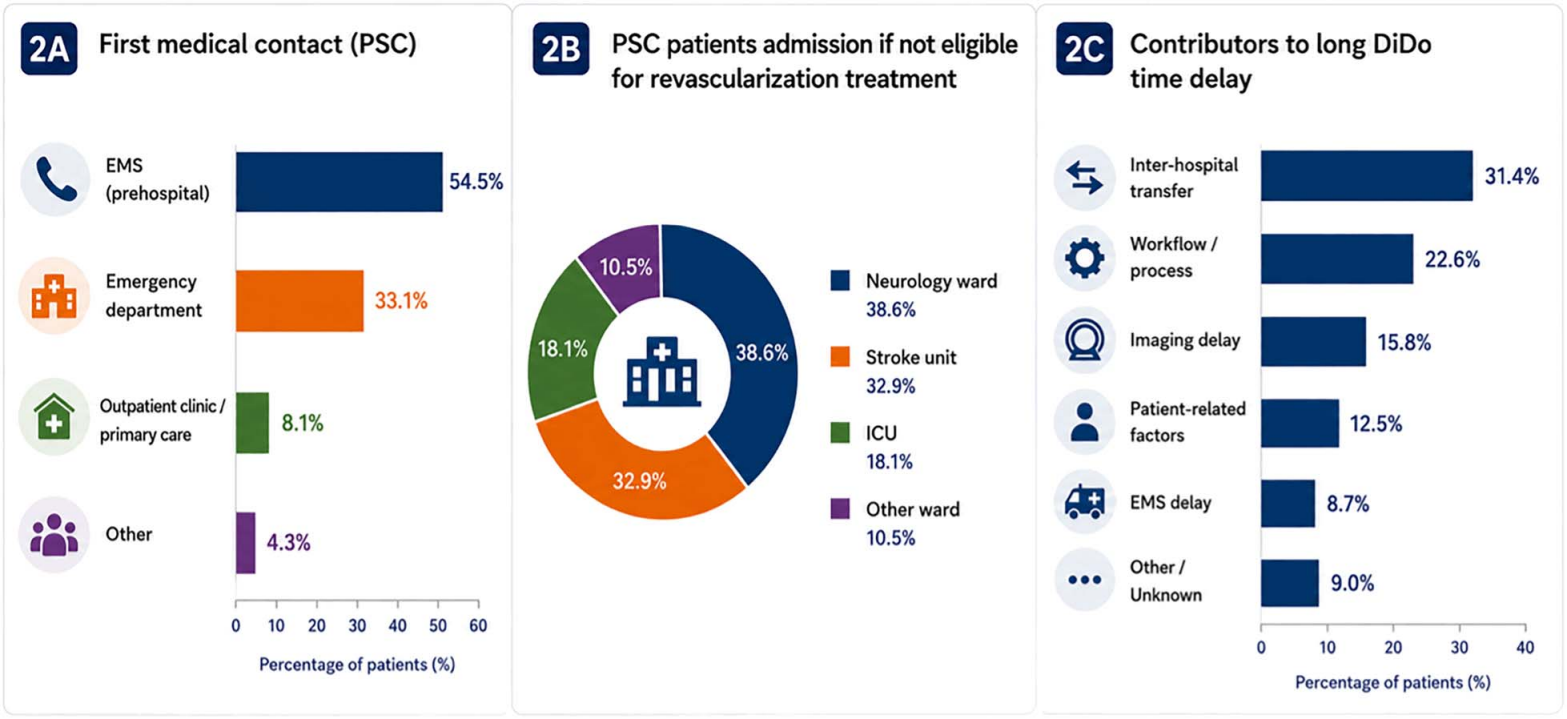
Primary stroke centre management and contributors to prolonged door-in–door-out time. Panel A shows the first medical contact for patients managed at primary stroke centres; panel B reports the admission destination for patients not eligible for revascularisation treatment and panel C shows the main contributors to prolonged door-in–door-out time. Abbreviations: DiDo = door-in–door-out time; EMS = emergency medical services; ICU = intensive care unit; PSC = primary stroke centre.

For patients with suspected LVO, 38.3% of PSCs recommended direct transfer to an endovascular-capable centre, while 61.7% did not. Regardless of symptom severity, patients were admitted primarily to the emergency department (86.7% for mild to moderate, 83.3% for severe). A dedicated stroke team conducted the initial evaluation in 51.7% of cases (Figure 2A), although this team was not available during nights and weekends in 30% of centres.

For patients ineligible for IVT/EVT, 58.3% were admitted to the emergency department, 26.7% directly to the stroke unit and 10% to the imaging unit (Figure 2B). Door-in–door-out time was not monitored in 25% of PSCs, and only 65% reported performing audits. Key delays were attributed to call-to-departure time (63.3%), imaging end-to-CSC call time (40%) and arrival-to-imaging start time (25%) (Figure 2C).

When patients were transferred to a CSC for thrombectomy, a physician was present in 68.3% of cases if IVT was ongoing. Otherwise, 50% were transported without a physician.

### Comprehensive stroke centres (CSCs)

The majority of responses (*n* = 185, 62.5%) came from CSCs. The EMS call-to-hospital arrival time was systematically monitored in 53.5% of centres, but follow-up audits were conducted in only 31.9%.

Regardless of symptom severity, patients were most frequently admitted to the emergency room (77.8% for mild to moderate symptoms, 73.5% for severe). Direct imaging access was reported in 16.8% (mild/moderate) and 18.4% (severe). A “code stroke” protocol was present in 81.1% of CSCs.

A dedicated stroke team performed the initial medical evaluation in 65.4% of cases, while the emergency room team performed it in 29.2%. The neuro-intervention team was not available on-site during nights or weekends in 42.2% of cases.

Admission-to-reperfusion time for patients undergoing thrombectomy was monitored in 90.8% of centres, but only 28.6% of centres conducted audits when delays occurred. For transferred patients with LVO, 41.1% were admitted through the emergency department, 35.7% had direct access to the angio-suite and 14.6% to the imaging unit. Transfer time from the outside hospital to CSC was not monitored in 39.5% of cases, with audits conducted in only 37.8%.

## Discussion

Our analysis shows substantial variability in prehospital stroke care across Europe. Stroke code protocols are widely implemented, and the priority of rapid reperfusion is well recognised. However, important shortcomings continue in prehospital assessment, triage, time monitoring and audit practices. These limitations may delay access to reperfusion therapies. More than 90% of centres reported activating a stroke code and pre-notifying the receiving hospital, but monitoring was inconsistent: 25.5% of services did not track EMS call-to-hospital arrival time. Transport destination decisions varied widely, with patients being sent to PSCs, CSCs or, in some cases, to the nearest hospital without reperfusion capability.

In this context, data from the Catalan Stroke Code registry provide complementary real-world evidence on EMS performance.^10^ In that cohort, EMS correctly activated the stroke code in 22,968 cases, accounting for 81% of ambulance transports. Patients for whom the stroke code was not activated more often presented with milder or atypical symptoms. These patients had lower NIHSS scores, longer onset-to-door times and a higher prevalence of posterior circulation strokes, which are less readily detected by standard FAST-based screening tools. Large vessel occlusion was less common in this group compared with patients receiving EMS stroke code activation (18% vs 24%).^10^

Magnusson et al. documented an EMS stroke recognition rate of 80.4% in a Swedish prehospital setting,^13^ whereas data from England reported that only 62% of patients identified as suspected stroke by ambulance clinicians were ultimately confirmed to have stroke or transient ischaemic attack.^14^

Important in-hospital organisational gaps were also evident beyond EMS identification. Only half of PSCs tracked EMS-to-hospital arrival times. Furthermore, many centres lacked 24/7 access to on-site dedicated stroke teams; 30% of PSCs reported that these teams were unavailable at night and on weekends. Data from the Swiss Stroke Registry show that patients with acute ischaemic stroke arriving at PSCs during night hours face higher odds of poor outcome and increased mortality.^8^ Door-in–door-out delays often resulted from long call-to-departure times, time lag between imaging and CSC calls and delays starting imaging.^8^ Our survey showed similar trends: call-to-departure time was the main source of delay (63.3%), followed by imaging end-to-CSC call (40%) and arrival-to-imaging start time (25%). In contrast, in a post-hoc analysis of the RACECAT trial, among patients enrolled during night-time direct transport to a thrombectomy-capable centre was associated with lower degrees of disability at 90 days.^15^

Regular clinical audits represent a cornerstone of quality assurance and improvement in stroke care. They allow continuous monitoring of key performance indicators such as onset-to-needle and door-to-groin times, identification of workflow bottlenecks and benchmarking of outcomes across centres.

Despite their proven effectiveness—audited centres have reported up to a 25% reduction in mortality and improved adherence to guideline-recommended therapies implementation remains limited, with fewer than one-third of CSCs and PSCs performing them routinely.^16^ Prioritising the expansion of audit participation and ensuring feedback-driven process improvement can enhance uniformity and overall performance within stroke networks.

In this setting, structured quality-improvement initiatives such as the EMS Angels Awards and national quality registers provide an important framework to address deficiencies in monitoring and benchmarking of prehospital stroke care.^17^

In addition to challenges with EMS identification, the survey identified substantial organisational vulnerabilities in hospital workflows. These gaps were pronounced at night and on weekends. Only half of PSCs systematically monitored EMS-to-hospital arrival times. Meanwhile, 30% reported the absence of dedicated stroke teams during off-hours. Furthermore, 42.2% of CSCs lacked on-site neuro-interventional team coverage during nights and weekends. These deficiencies are likely to increase treatment delays during off-hours. They may account for the poorer outcomes observed in registry-based studies.

Although the ESO survey did not directly compare treatment metrics between daytime and nighttime, it identified several workflow steps that are especially vulnerable during off-hours. Door-in–door-out delays were most often attributed to prolonged call-to-departure times (63.3%).

Additional causes included delays between imaging completion and CSC notification (40%) and delays in initiating imaging (25%). Reduced staffing in emergency and radiology departments likely contributes to these extended door-to-needle times. Dependence on on-call neurologists for IVT decisions at night is another factor.^18^ Moreover, the volume of neuro-intervention procedures has increased in recent years and so have neuro-intervention consultations. Data reported the highest percentage of consults leading to thrombectomy occurring in the early morning hours after midnight (87% of consultations between 00:00 and 04:00 AM proceeding to thrombectomy compared to 37% between the hours of 04:00 PM and 08:00 PM)^19^ with a mean of consultations and performed procedures per day of 0.79 and 0.32 (1 every 3 days), respectively^20^ but neuro-intervention teams are not always available in CSCs. Only 42.2% are available during night hours or weekends, the median time from imaging completion to procedural start is about an hour longer during non-work hours than work hours with a median physician time spent on NT responsibilities per 24-h call period of 69 min.^21^

Lastly, interhospital transfers to CSC often did not include a physician, unless IVT was in progress. As many European stroke networks switch from alteplase to tenecteplase (TNK), this may change. Tenecteplase’s single dose, longer action and lower risk of transport issues make it well-suited for patients who need transfer in DS model. Unlike alteplase, TNK does not need continuous infusion or pump management, which may reduce the need for a physician during transfer and help prevent treatment delays.^22^

This study has some limitations. We acknowledge the relatively low response rate. Of approximately 4000 invited participants, 296 responded, resulting in a response rate of about 6.2%. This may have introduced non-response and self-selection bias, which should be considered when interpreting the representativeness and generalisability of the findings. However, this response rate aligns with known challenges in physician web-based survey research, where participation is often limited by survey burden and time constraints, as previously reported in studies of physician specialists.^23^

Moreover, we have limited data from non-urban networks, as most comes from urban areas. Rural areas have completely different logistics than city centres, so more data from non-urban areas are needed, as a single pre-hospital model may not be suitable for every environmental situation. Second, reliance on reports from centres introduces possible recall and reporting bias (eg, regarding monitoring, protocols, staffing, which differ between CSC and PSC). Third, there was considerable heterogeneity in the data across European countries. We received many responses from countries such as Spain, Italy and the Netherlands, but relatively few from Germany, Belgium, Austria, Switzerland and others. This imbalance may help explain why MSU appeared to be universally unavailable in our survey. Finally, data collection is survey-based and self-reported.

A further limitation is the relatively low proportion of EMS respondents. Although the first author is an emergency physician and EMS physicians from the ANGELS EMS network, who are actively involved in stroke care, were specifically targeted, their participation remained limited.^11^ This may have reduced the representativeness of the prehospital perspective, although similar response patterns have been observed in previous broad web-based surveys of healthcare professionals.^23^ Additionally, EMS organisation differs substantially across countries, including dispatcher systems, first-responder models, ambulance staffing and prehospital triage pathways. Although the survey captured selected aspects of EMS practice, it was not designed to provide a detailed country-level characterisation of EMS structures; therefore, these differences may have influenced the reported transport patterns and limit the comparability of prehospital responses across countries.

Finally, although the study database was reviewed by authorised study team members to identify potential duplicate or inconsistent entries, the lack of a formal technical mechanism restricting participation to one respondent per institution means that multiple responses from the same centre cannot be entirely excluded, potentially resulting in within-centre correlation.

A principal strength of this study is its evaluation of auditing and time-interval monitoring practices throughout prehospital and early in-hospital stroke care pathways in several European countries. The findings reveal significant deficiencies in the implementation of routine audits. Stroke code activations are common in this setting. The study identifies a crucial and modifiable system-level weakness that may contribute to treatment delays. This emphasis offers practical recommendations to standardise performance monitoring and enhance reperfusion delivery within stroke networks.

## Conclusions

Despite the widespread adoption of stroke code activation across Europe, significant gaps remain in time monitoring, auditing and off-hours organisation throughout the stroke care pathway. These shortcomings may contribute to preventable treatment delays. Enhancing systematic auditing and quality improvement frameworks within EMS and hospital settings is necessary and needs to be in place as part of the stroke chain of recovery.

## Supplementary Material

Supplemental_Material_clean_aakag113
