# Supplementary material for: European Stroke Organisation survey on reperfusion therapy practices
Source: Eur Stroke J. 2026 Sep 18;11(9):aakag113. doi: 10.1093/esj/aakag113 (PMC13588096; doi:10.1093/esj/aakag113)
Supplement: Supplementary file 1 [file supplemental_material_clean_aakag113.docx]

**Supplemental Material**

***Figure S1: e-survey***

**Questionnaire to all participants:**

**Q1: In which European country do you work?**

🡪 List of European countries (WHO list, which includes Israël)

**Q2: What is your professional status?**

- Paramedic/ambulance nurse
- Stroke nurse
- Neurologist
- Stroke physician
- Neuro-interventionalist
- Emergency room physician
- Other: notify

**Q3: In which environment do you work?**

- Urban area (city center)
- Suburban area
- Rural area

**Q4: In which setting do you work?**

- EMS (emergency medical service)
- Primary stroke center (i.e., no endovascular treatment capability)
- Comprehensive stroke center (i.e., endovascular treatment capability)

The answer to Q4 leads to different subsequent questionnaires.

**Questionnaire for EMS staff:**

**Situation:** You receive a call from a patient (or her/his relatives) who has symptoms suggestive of a stroke.

**Q1:** What type of transporter usually drives to the patient?

- Transporter with first-aid provider only (i.e., without paramedic or physician)
- Transporter with paramedic or ambulance nurse only (i.e., without physician)
- Transporter including a physician
- Mobile stroke unit (i.e., also including CT-scanner and point of care laboratory)

**Q2:** Do you use a pre-hospital scale for stroke identification and/or severity graduation?

- Yes
- No

If yes: which scale(s)?

**Situation:** The patient indeed has a suspected acute stroke

**Q3:** Do you have a ‘code stroke' protocol?

- Yes
- No

**Q4:** Do you systematically pre-notify the target hospital for suspected acute stroke?

- Yes
- No

**Q5:** Do you use ambulance telemedicine for patient’s triage?

- Yes
- No

**Q6:** To which type of hospital do you usually transport a patient with a suspected acute stroke with mild to moderate symptoms?

- nearest hospital, regardless of whether this hospital can provide reperfusion therapy (thrombolysis or thrombectomy)
- nearest hospital with ability to provide intravenous thrombolysis
- nearest hospital with ability to provide endovascular thrombectomy

**Q7:** To which type of hospital do you usually transport a patient with a suspected acute stroke with severe symptoms?

- nearest hospital, regardless of whether this hospital can provide reperfusion therapy (thrombolysis or thrombectomy)
- nearest hospital with ability to provide intravenous thrombolysis
- nearest hospital with ability to provide endovascular thrombectomy

**Q8:** Where in the hospital do you usually drop-off the patient with mild to moderate symptoms?

- Emergency room
- Directly in the imaging unit
- Directly in the stroke unit
- Directly in the angio suite
- Other: notify

**Q9:** Where in the hospital do you usually drop-off the patient with severe symptoms?

- Emergency room
- Directly in the imaging unit
- Directly in the stroke unit
- Directly in the angio suite
- Other: notify

**Q10:** Where in the hospital do you usually drop-off the patient not eligible for revascularization treatment

- Emergency room
- Directly in the imaging unit
- Directly in the stroke unit
- Other: notify

**Q11:** Do you systematically monitor the first EMS call-to-hospital arrival time delay?

- Yes
- No

**Q12:** Do you regularly make an audit of the first EMS call-to-hospital arrival time delay?

- Yes
- No

**Q13:** Do you share the first EMS call-to-hospital arrival time delay with the hospital?

- Yes, manually
- Yes, electronically
- No

**Q14:** Do you systematically manage the patient not eligible for revascularization treatment as fast as the patient with acute stroke?

- Yes
- No

**Questionnaire for Primary Stroke Center:**

**Situation:** You receive a call from EMS (emergency medical service) for an acute stroke suspicion

**Q1:** Do you systematically monitor the EMS call-to-hospital arrival time delay?

- Yes or no

**Q2:** Do you regularly make an audit of the EMS call-to-hospital arrival time delay in your center?

- Yes
- No

**Q3**: Do you have a ‘code stroke’ protocol in case of acute stroke suspicion pre-notified by EMS?

- Yes or no

**Q4:** In case of suspicion of large vessel occlusion ischemic stroke (eg, severe symptoms described by the EMS team), do you recommend to directly transfer the patient to the nearest endovascular capable center?

- Yes or no

**Q5:** Where are the patients with mild to moderate symptoms (as described by the EMS team) first admitted in your hospital?

- Emergency room
- Directly to imaging
- Directly to the stroke unit
- Other: notify

**Q6:** Where are the patients with severe symptoms (as described by the EMS team) first admitted in your hospital?

- Emergency room
- Directly to imaging
- Directly to the stroke unit
- Other: notify

**Q7:** Who usually first takes care of acute stroke suspicion in your hospital?

- Emergency room team
- Dedicated stroke team
- Emergency room team + telestroke physician
- Other: notify

**Q8**: Is the staff (physicians, nurses, etc…) who takes care of acute stroke patients is present on site also during night hours or weekend?

- Yes
- No

**Q9:** Where in the hospital is usually admitted the patient not eligible for revascularization treatment

- Emergency room
- Directly in the imaging unit
- Directly in the stroke unit
- ICU
- Other: notify

**Situation:** The patient has an acute ischemic stroke with a large vessel occlusion, with indication for endovascular treatment.

**Q 10:** Do you systematically monitor the door in- (arrival in your center) to-door out (departure to the endovascular capable center) time delay?

- Yes
- No

**Q11:** Do you regularly make an audit of the door in-to-door out time in your center?

- Yes
- No

**Q12:** In your opinion, what are the main contributors to long door in-to-door out time delay (class them from the most important to the less important contributor)?

Hospital arrival to imaging start

Imaging time

Imaging end to telestroke evaluation

Imaging end to endovascular capable center call

Endovascular capable center call to departure

Others

**Q13:** What type of transporter usually transfers the patient while treated with intravenous thrombolysis?

- Transporter with first-aid provider only (i.e., without paramedic or physician)
- Transporter with paramedic or ambulance nurse only (i.e., without physician)
- Transporter including a physician

**Q14:** What type of transporter usually transfers the patient NOT treated with intravenous thrombolysis?

- Transporter with first-aid provider only (i.e., without paramedic or physician)
- Transporter with paramedic or ambulance nurse only (i.e., without physician)
- Transporter including a physician

**Questionnaire for Comprehensive Stroke Center:**

**Situation:** You receive a call from EMS (emergency medical service) for an acute stroke suspicion

**Q1:** Do you systematically monitor the EMS call-to-hospital arrival time delay?

- Yes
- No

**Q2:** Do you regularly make an audit of the EMS call-to-hospital arrival time delay in your center?

- Yes
- No

**Q3**: Do you have a ‘code stroke’ protocol in case of acute stroke suspicion pre-notified by EMS?

- Yes
- No

**Q4:** Where are the patients with mild to moderate symptoms first admitted in your hospital (this does not concern patients transferred from an outside hospital)?

- Emergency room
- Directly to the imaging unit
- Directly to the angio-suite
- Directly to the stroke unit
- Other: notify

**Q5:** Where are the patients with severe symptoms first admitted in your hospital (this does not concern patients transferred from an outside hospital)?

- Emergency room
- Directly to the imaging unit
- Directly to the angio-suite
- Directly to the stroke unit
- Other: notify

**Q6:** Who usually first takes care of acute stroke suspicion in your hospital?

- Emergency room team
- Dedicated stroke team
- Other: notify

**Q7**: Is all the staff (including the neuro-intervention team) who takes care of acute stroke patients with large vessel occlusion present on site also during night hours or weekend?

- Yes
- No

**Q8:** For ischemic stroke patients treated with thrombectomy, do you systematically monitor admission to reperfusion (ATR) time delay?

- Yes
- No

**Q9:** For ischemic stroke patients treated with thrombectomy, do you regularly make an audit of the admission to reperfusion (ATR) time delay in your center?

- Yes
- No

**Situation:** You receive a call from an outside hospital for an acute ischemic stroke with large vessel occlusion

**Q10:** Do you monitor the outside hospital call to your hospital arrival time delay?

- Yes
- No

**Q11:** Do you regularly make an audit of the outside hospital call to your hospital arrival time delay?

- Yes
- No

**Q12:** Where do you usually admit the patient after the inter-hospital transfer?

- Emergency room
- Directly to the imaging unit (for repeat or complete imaging)
- Directly to the angio suite
- Directly in the stroke unit
- Other: notify
